# Acute presentations of intradural lipomas: case reports and a review of the literature

**DOI:** 10.1186/s12883-019-1413-4

**Published:** 2019-08-08

**Authors:** Luca Massimi, Thailane Maria Feitosa Chaves, François Yves Legninda Sop, Paolo Frassanito, Gianpiero Tamburrini, Massimo Caldarelli

**Affiliations:** 1 Pediatric Neurosurgery - Neurosurgery Department, Fondazione Policlinico Universitario A. Gemelli IRCCS, Largo A. Gemelli, 8, 00168 Rome, Italy; 2 Università Cattolica del Sacro Cuore, Istituto di Neurochirurgia, Roma, Italy; 3 Departamento de Neurocirurgia Pediátrica, Casa de Saúde Santa Marcelina, Sao Paulo, Brazil

**Keywords:** Spina bifida occulta, Spinal lipoma, Natural history, Surgical indications, Prophylactic surgery

## Abstract

**Background:**

Lumbosacral lipomas (LLs) may remain asymptomatic or lead to progressive neurological deterioration. However, sudden neurological deterioration is a rare and severe event. Herein, we report rare occurrences of sudden clinical deterioration in two previously asymptomatic children harbouring intradural LLs without dermal sinus tracts or signs of occult dysraphism. A review of the pertinent literature is also included.

**Case presentation:**

One child exhibited acute deterioration because of an epidural abscess associated with a filar lipoma without a sinus tract (probably caused by haematogenous spreading from a respiratory tract multiple infection), and the other child exhibited acute deterioration because of a very large, holocord syringomyelia-like cyst associated with a small conus lipoma. Both patients were 4 years old. In case #2, a previously undetected, severe tethered cord (conus at the S3-S4 level) was also present. A complete recovery was attained after an urgent surgical operation in both cases (in addition to targeted antibiotic therapy in case #1). All cases of deterioration in the literature were caused by abscess formation in dermal sinus tracts.

**Conclusions:**

Prophylactic surgery may be indicated even in asymptomatic children that have tethered cord and surgically favourable LLs (small dorsal and filar LLs), especially if the conditions are associated with progressive syringomyelia. Similarly, intradural dermal sinus tracts should be regarded as surgery-indicated, even if the conus is in its normal position and the patient is asymptomatic because there is a consistent risk of severe, infection-related complications. Finally, asymptomatic patients with filar LLs and a normally located conus can be candidates for surgery or an accurate clinical and radiological follow-up.

## Background

Lumbosacral lipomas (LLs) usually accompany occult spinal dysraphism and are diagnosed in neonates/infants based on skin markers; they are occasionally diagnosed in children/young adults based on clinical deterioration [1, 2]. LLs account for 1% of all spinal masses and present as intradural lesions without spinal bifida in approximately 25% of cases [3–5]. Several classifications have been proposed so far to contextualize LLs, including that provided by Tortori Donati et al.: LLs without dural defects and LLs with dural defects [5]; Arai et al.: filar, caudal, dorsal, combined or transitional lipomas and lipomyelomeningoceles [1]; Oi et al.: spinal lipomas without meningoceles, lipomeningoceles, lipomyelomeningocystoceles and lipomyelomenongoceles [6]; and Pang D: dorsal transitional, terminal and chaotic lipomas [7]. Patients are usually asymptomatic at presentation or show progressive neurological deterioration because of the tethering of the spinal cord. The main clinical manifestations are back and leg pain, motor and/or sensory deficits, and bladder and/or bowel dysfunction. Acute neurological deterioration is rarely described and is usually attributable to the abscessation of dermal sinus tracts [8–12].

Herein, we report rare occurrences of sudden clinical deterioration in two previously asymptomatic children without signs of occult dysraphism. The goal is to describe a new possible clinical presentation of intradural LLs and to discuss the well-known dilemma of whether to operate on asymptomatic patients. In addition, the most recent and pertinent literature has been analysed.

## Case presentation

### Case #1

A 4-year-old boy was referred to our unit in August 2017 from another hospital because he developed sudden left lumbar cruralgia after a moderate back injury that occurred 2 weeks prior during a recreational activity. The child also had a fever, which started almost simultaneously with the head injury. The past medical history was unremarkable (as well as the familial and the psychosocial history), with the exception of frequent episodes of respiratory tract infections.

**Fig. 1:**
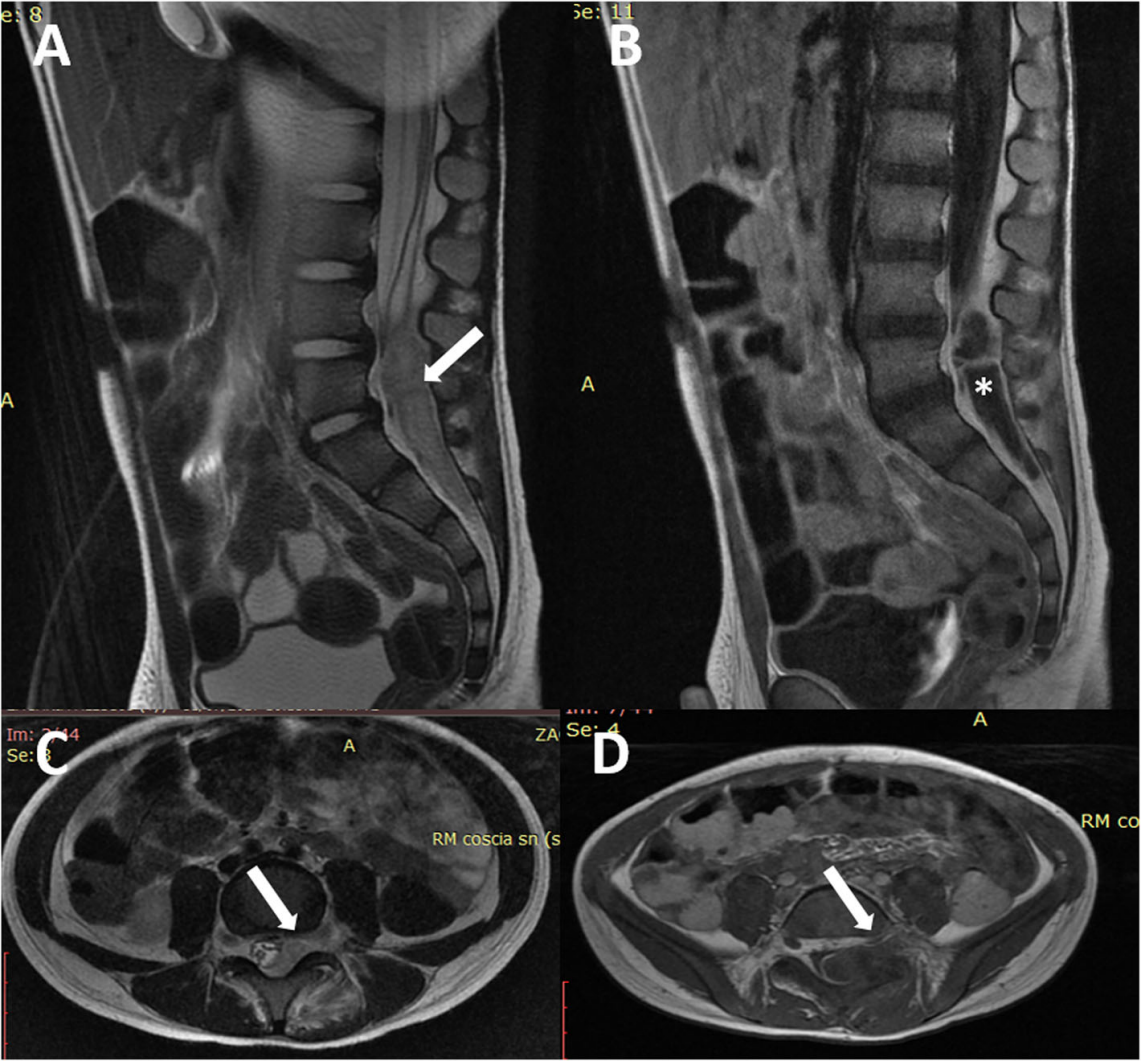
Preoperative MRI scan of case #1. An epidural abscess (L4-S2) is evident on sagittal T2w (**a**, arrow) and gadolinium T1w (**b**, asterisk). The respective axial images (**c**, **d)** show the compression on the dural sac and the involvement of the left L4-L5 foramen (arrow)

At the physical examination, the child was conscious and was complaining of lumbar pain radiating to the anterior thigh, palpation of the lumbar spine evoked the pain, no stiff neck was present, no skin markers were detected, and his body temperature was 38.5 °C. The neurological examination revealed no motor or sensory deficits, bladder disorders, or bowel disorders; however, the patient could not walk because of the intense pain. Once admitted, the child underwent spinal cord magnetic resonance imaging (MRI), which showed an intra- and extradural lesion extending from the lower L4 vertebra to the S2 vertebra, resulting in compression of the medullary conus and roots. The lesion appeared to be a fluid collection, with contrast enhancement, similar to an abscess (Fig. 1). Blood leucocytosis and increased levels of inflammation markers were detected.

The day after admission, a surgical excision of the lesion was performed through a L5-S1 laminectomy. A purulent collection that filled the epidural space was completely removed and sent for microbiological examination. At the S1 level, a partially collapsed lipoma of the filum that occupied the subdural space was progressively separated from the nerve roots under neurophysiological monitoring and excised by sectioning the terminal filum. The procedure was completed by duraplasty. The L5-S1 laminae were not replaced in order to leave the spinal cord decompressed. The regeneration properties of the bone at this age and the static behaviour of the sacral vertebrae are likely to close the bony gap, avoiding instability problems.

A histological analysis of the surgical samples confirmed the diagnosis of a lipoma.

**Fig. 2:**
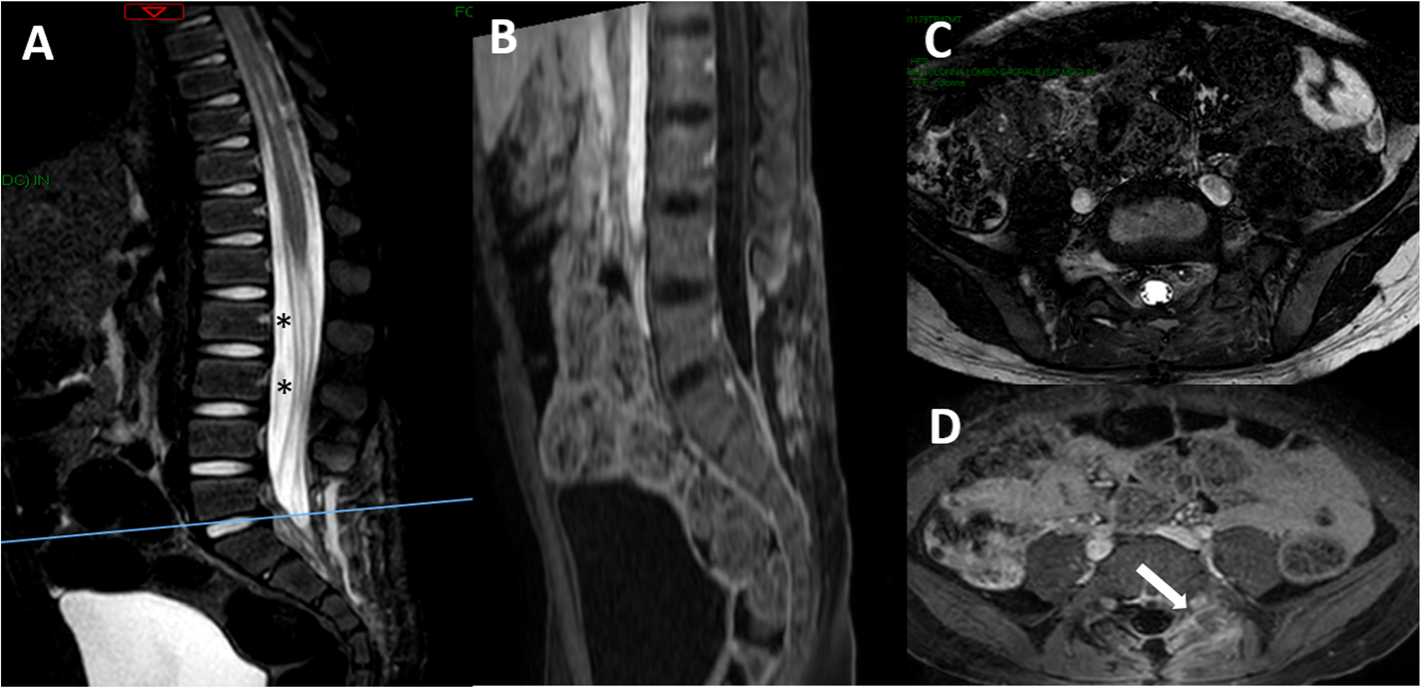
Sagittal (**a**) and axial T2w (**c**) and sagittal (**b**) and axial gadolinium T1w MTI (**d**) showing the normalization of the picture of case #1 1 month after surgery and immediately after the antibiotic therapy. The caudal roots are clearly visible and decompressed (asterisks), and no tissue occupying the left L4-L5 foramen is visible (arrow)

The postoperative course was uneventful. The child showed a rapid recovery from the preoperative pain. The culture of the abscess revealed the presence of a methicillin-sensitive *Staphylococcus aureus*, so a targeted antibiotic therapy was carried out for 4 weeks. Postoperative MRI (performed 1 month later) showed a normalization of the radiological picture (Fig. 2). At the current follow-up (16 months), the child is asymptomatic.

### Case #2

A 4-year-old boy was referred to our unit in May 2018 from the Pediatric Intensive Care Unit where he was admitted 2 days prior with a suspected case of Guillain-Barré syndrome. The clinical history had started with urinary incontinence associated with paraparesis, which quickly progressed and prevented ambulation. The past familial, medical and psychosocial history was unremarkable.

**Fig. 3:**
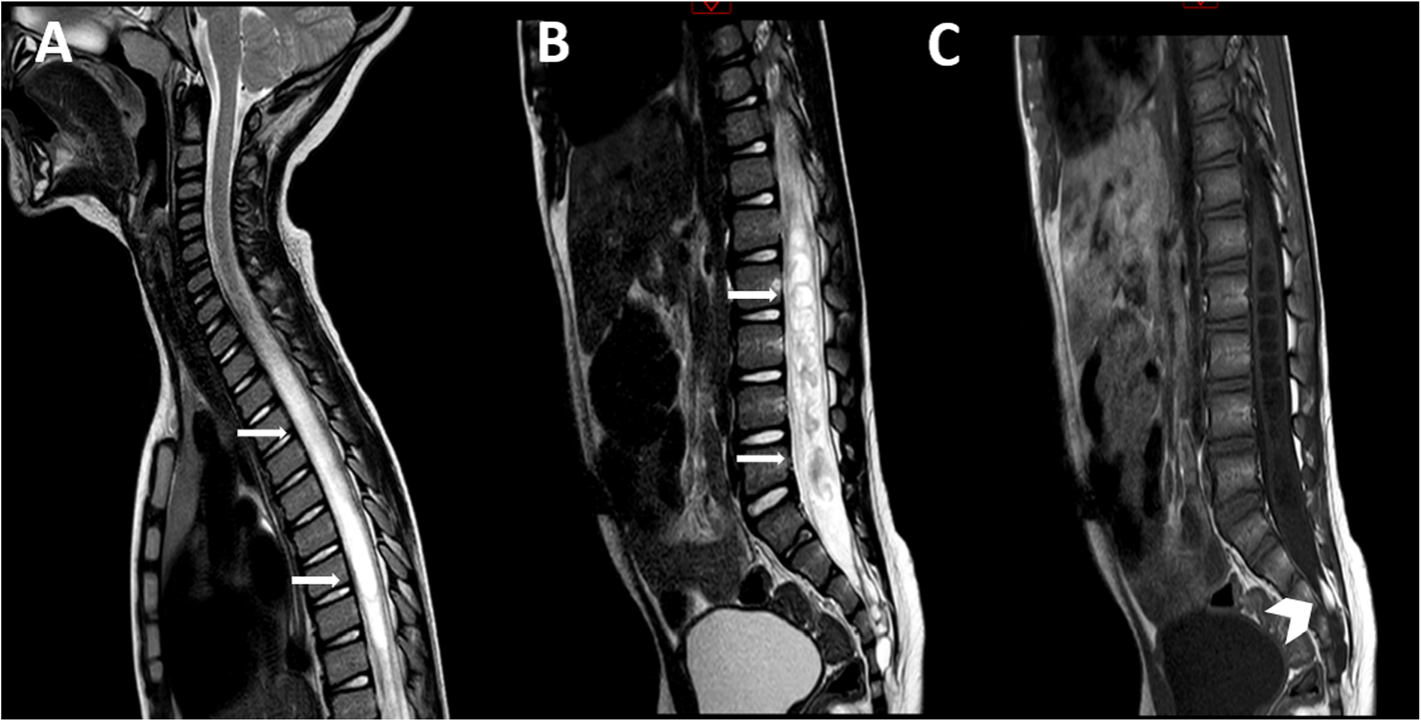
Sagittal T2 cervicodorsal (**a**) and lumbosacral MRI (**b**) of case #2 showing a very large dorsolumbar syringomyelia (arrows) and severe tethered cord, with the conus at S3-S4 level. The T1w sequence (**c**) demonstrates a small lipoma of the conus as a cause of tethering (head arrow)

At the time of admission, the patient was conscious; the neurological examination demonstrated paraparesis (2/5) with severe deficits in dorsiflexion of the feet, a moderate deficit in trunk elevation, diffuse hypoesthesia of the lower limbs and urinary incontinence. The MRI scan of the spinal cord (Fig. 3) documented a large dorsolumbar syringomyelia secondary to a severe tethered cord, supported by a small lipoma of the conus, which was stretched to reach the S3-S4 level.

The child underwent surgery immediately after the MRI scan. Through an L5-S4 laminectomy, the dural sac was opened to explore and decompress the spinal cord. The small distal lipoma was dissected and removed, which immediately detethered the spinal cord.

The diagnosis of a lipoma was confirmed by histological examination.

**Fig. 4:**
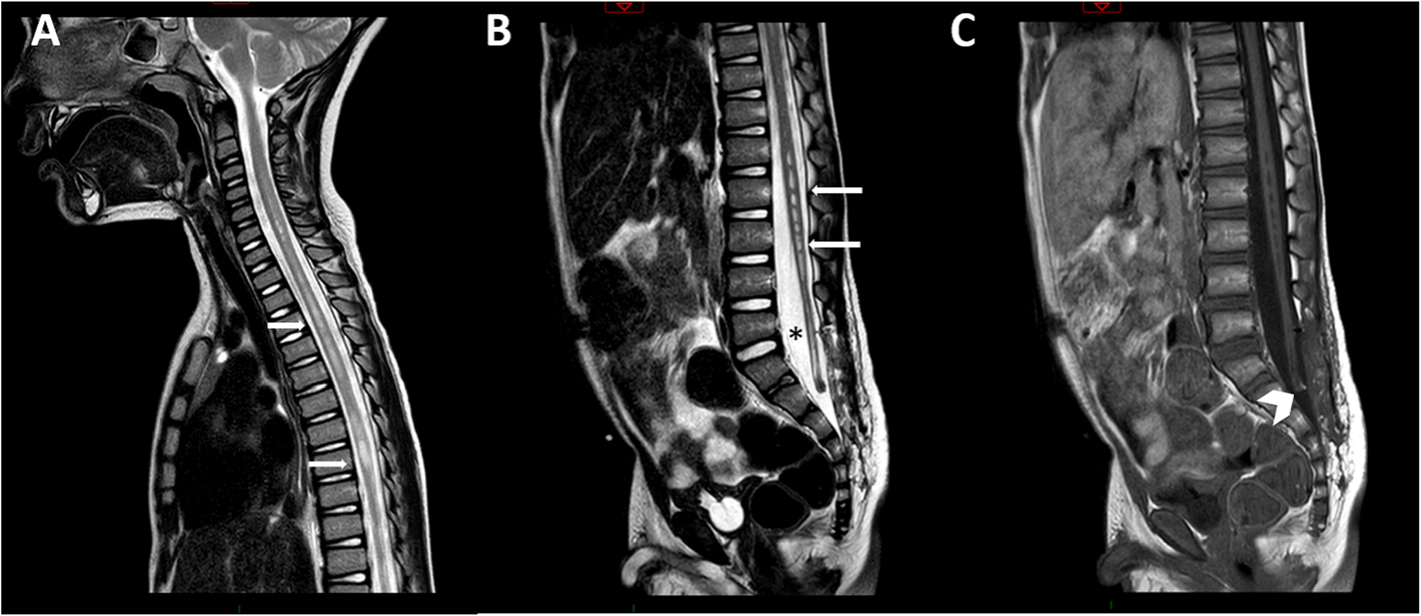
MRI scan of case #2 performed 3 months after surgery. The detethered spinal cord is now floating inside the dural sac (asterisk). The syringomyelia is significantly reduced on the sagittal T2 cervicodorsal (**a**, arrows) and lumbosacral MRI (**b**, arrows) scans. A very small remnant of the lipoma can be seen on the T1w sequence (**c**, head arrow)

The postoperative course was uneventful. The child showed a progressive improvement in both paraparesis and urinary incontinence, which were normalized after 3 weeks. The hypoesthesia significantly improved but still persisted in the 7-month follow-up. The postoperative MRI scan, performed 3 months after surgery, showed the detethering of the spinal cord and the significant reduction of the syringomyelia (Fig. 4).

## Discussion and conclusions

### Controversies on the management

The management of asymptomatic children with LLs remains controversial. Some authors advocate routine surgery for all patients, regardless of the presence of symptoms or the patient’s age, to prevent neurological deterioration [13, 14]. For example, Pang et al., based on the favourable long-term outcomes of patients with asymptomatic congenital LLs that underwent total surgical resection (98.4% progression-free survival at 16 years), strongly support prophylactic surgery [15, 16]. Moreover, Oi et al. found that the neurological improvement of symptomatic patients after surgery is not optimal and that the rate of surgical complications is acceptably low, providing two additional arguments in favour of the “prophylactic strategy” 6. On the other hand, other authors are against prophylactic surgery, based on the possible surgical risks and the analysis of the natural history of LLs, which demonstrates a significant rate of patients do not present deterioration over time (up to 70%) [17–19]. In addition, there is a third option that takes into account the types of LLs. Consequently, surgery is indicated for patients with LLs with a “favourable” surgical anatomy, such as caudal, filar and small dorsal types of LLs (where the surgical excision is usually easy and the risk of deterioration is higher than the risk of surgical complications), and it is indicated only for symptomatic patients with the less favourable types of LLs (transitional and chaotic lipomas) [1, 20, 21].

### Neurological deterioration

On these grounds, it is clear that the main factor influencing the indication for surgery is neurological deterioration, which should be avoided in asymptomatic cases and reverted in symptomatic cases. The present experience raises the problem of the prevention of neurological deterioration in cases where it occurs suddenly and/or the lipoma could not be detected in advance. Actually, both of our patients showed a quickly deteriorating clinical course without any previous first signs. Instead, neurological deterioration usually occurs slowly and progressively, providing time to make the diagnosis or prepare a treatment. The main mechanism of deterioration is related to chronic ischaemic damage due to spinal cord tethering [22]. The intermittent stretching forces exerted on the caudal spinal cord can cause hypoxemia and ischaemia followed by inhibition of the oxidative metabolism and electrical activity in the spinal cord [23]. Such a mechanism can be worsened by flexion movements of the spinal column that cause “lengthening” of the vertebrae even with a thick terminal filum and no lipoma [24]. Additional factors that induce deterioration can include effects of the lipoma mass (compression on the conus and on the roots of the cauda equina) [20] and/or an associated syringomyelia [22]; alternatively, deterioration could be induced by a possibly associated myelodysplasia, but this hypothesis is not accepted by all authors [25]. According to their study on the natural history of asymptomatic cases, Wykes et al. found an age < 2 years, the female sex, the transitional type of lipoma and the presence of syringomyelia correlate with a high risk of deterioration (40% cumulative risk 10 years after the diagnosis) [19]. The aforementioned findings explain the “usual” chronic deterioration, occurring either early or late in the clinical history of the patients. Instead, both of our cases presented rapid deterioration, with root compression in case #1 (by an abscess occupying the epidural space) and a relevant tethered cord in case #2. These findings could be basal pathological conditions causing mechanical stress on the spinal cord, precipitated by the occurrence of associated factors.

### Case report considerations

In case #1, the associated factor was a previous mild/moderate back injury. Therefore, one could speculate on the presence of a silent abscess (favoured by multiple respiratory infections) decompensated by the trauma. The abscess formation in an occult dysraphism is one of the most common precipitating factors reported in the literature (Table 1). It usually occurs as a result of an infection of a dermal sinus tract, where the interaction between the neural tissue and the external environment provides an obvious source of infection. In rare cases, as in the present case, such an interaction is not evident. This very uncommon phenomenon can be explained by the haematogenous spreading of an infectious process [26]. The lipoma acts as a germ growth pabulum in cases of asymptomatic, transient bacteraemia caused by dental procedures, subclinical infections, etc [27, 28] The composition of the lipoma (tissues originating from all three germ layers) can increase the risk of haematogenous spreading. Moreover, in some lipomas with presacral extension (approximately 6.5% of cases), the infection could result simply from being in close proximity with the rectum [20]. In case #1, the trauma could also have played a role. The direct transfer of traumatic forces to a stretched spinal cord through the lipoma (by flexion injury, abrupt disc herniation, etc.), is an obvious and accepted mechanism of sudden deterioration [29]. However, the exact role of the injury in deterioration as well as the incidence of the phenomenon cannot be established exactly. On the other hand, case #2 showed a large syringomyelia as a co-factor for quick deterioration. The presence of terminal hydromyelia or syringomyelia in LL is quite common, with an incidence varying from 2.5 to 10% [30, 31]. The role of the syrinx in the patient’s symptomatology is unclear, although its occurrence and progression are considered an indication for surgery by many neurosurgeons in daily clinical practice. Additionally, the mechanism of syrinx formation is not yet clear, since it can occur even after spinal cord detethering [1, 32].

**Table 1:** Synopsis of the most recent cases of sudden deterioration

Author, year	No. cases	Age	Type of lesion	Deterioration	Treatment	Late outcome
Vadivelu et al., 2014 [12]	2	17 mts, 26 mts	Undiagnosed dermal sinus + dermoid cyst + syrinx Undiagnosed dermal sinus + dermoid cyst	Intramedullary abscess with motor/sphincter deficit Intramedullary abscess with recurrent meningitis and hydrocephalus	Surgery + antibiotic therapy	Assistive device for walking Developmental delay + VP shunt
Bhanage et al., 2015 [8]	1	4 mts	Dermal sinus and tethered filum terminale	Leg weakness and infection (dorsal and lumbosacral intramedullary abscess (D11-S3)	Surgery + antibiotic therapy (*Mycobacterium tuberculosis)*	Left foot deformity with limping gait and a neurogenic bladder
Singh et al., 2015 [11]	3 (14.2%) out of a series of 21 cases	9 mts to 15 yrs. (mean: 8.2 yrs)	Dermal sinus	Meningitis, intraspinal abscess and acute paraplegia	Surgery + antibiotic therapy	Persistent neurological deficit in one case, persistent sphincter deficit in all 3 cases
Girishan et al., 2016 [9]	10	8 to 24 mts (one case: 25 yrs)	Dermal sinus + intramedullary dermoid cyst	Rapid onset paraparesis secondary to infection (9 cases) or rupture of dermoid cyst in one case (quadriparesis)	Surgery + medical treatment (9 cases), only medical treatment (1 case)	Significant neurological improvement (8 cases), stable deficits (1 case), death (1 case, no surgery)
Rashid et al., 2016 [10]	1	2 yrs.	Dermal sinus	Infection (myelitis/polyradiculitis) with full motor/sensitive/sphincter deficit	Surgery + antibiotic therapy	Permanent neurological deficits

Independent of its causes, sudden neurological deterioration in patients with previously asymptomatic LL remains a rare but often severe and well-recognizable event. In case #2, the child experienced a quickly progressing paraparesis with bladder dysfunction. In cases of abscess formation, the clinical picture is even more severe because of the complications resulting from meningitis/myelitis, and the patients often show persistent deficits at a later follow-up (Table 1). To date, according to the recent review of the literature provided by Prasad et al., 50 cases of intramedullary abscess (mainly by *Staphylococcus aureus*) secondary to dermal sinus tracts (with the inclusion of cysts in 50% of cases) have been reported in children; a good-to-excellent outcome was observed in only 60% of cases, and a significant mortality rate was reported (8%) [33]. A fever and limb weakness were associated with a poor outcome, thus justifying the recommendation (stressed by all authors of the present study) of a timely surgical and medical treatment.

### How to prevent the risk of deterioration

On these grounds, two main issues seem to be relevant to minimizing the risk of neurological deterioration and improving its final outcome. The first issue is the diagnosis. To the best of our knowledge, we report here the first two cases of sudden deterioration in patients with truly “occult” LL (no symptoms, no dermal sinus tracts, no skin markers, no spina bifida), even though the presence of an abscessed epidural dermoid cyst cannot be theoretically excluded in case #1. Such a rare occurrence is the strength and, at the same time, the main limitation of this paper. As mentioned, the problem is not trivial because approximately one fourth of LL are without dural defects, though they can be associated with a tethered cord and/or syndrome [5]. Therefore, particular attention should be paid to premonitory symptoms in this subset of patients. Neurological, neuro-orthopaedic and sphincter-related signs are indeed crucial factors for the clinical diagnosis of LL, as they are the only revealing signs of an occult dysraphism in absence of skin markers. Unfortunately, these signs often occur subtly and/or progress slowly, so they may be underestimated and lead to a later diagnosis (mean age: 7 years) compared with the time of a diagnosis based on the presence of skin stigmata (mean age: 9 months) 20. Nevertheless, in many instances, the cutaneous stigmata is present, or the sinus tract is visible; however, these signs can be misdiagnosed [34]. Skin markers are actually found in 2.2 to 7.2% of all newborns, even though only a minority of them show an occult dysraphism and less than 1% require a surgical operation [35–37]. In a study of 439 newborns undergoing lumbosacral ultrasounds for a sacrococcygeal dimple (the most “innocent” among the skin markers), we demonstrated an abnormal pattern in 39 cases (8.8%), corresponding to an MRI-confirmed occult dysraphism in 12 cases (2.7%) [38]. These results prompted us to recommend routine ultrasounds in neonates with skin markers (followed by an MRI scan in suspected cases) to reduce the risk of a missed diagnosis. Finally, the risk of sudden deterioration can be decreased by a correct neuroradiological diagnosis. This diagnosis does not concern obvious lesions, such as LLs, but rather the dermal sinus tracts, which often appear to be extradural or both extraspinal and intradural. For example, the T2-weighted 3D pulse with drive MRI sequences has been preliminary found to significantly improve the view of the CSF/spinal cord/roots interface, thus enhancing the possibility of detecting the intradural course of a sinus tract [39]. This technique has been used thus far for the visualization of cranial nerves and/or neurovascular conflicts [40, 41].

### Asymptomatic patients

The second issue is the management of asymptomatic patients. The management of children with complex LLs (transitional and chaotic LLs) and tethered cord remains inconclusive. Indeed, this point remains largely controversial, and it is very difficult to summarize the advantages and disadvantages of surgery from the literature due to the accumulation of heterogeneous data from case series concerning different types of treated lipomas, rates of preoperative symptomatic patients (progressive versus non-progressive), and outcomes of the different symptoms (namely, urinary, orthopaedic, and neurological). Moreover, some authors are accustomed to operating on symptomatic patients only, while other authors include both asymptomatic and symptomatic patients in their series, preventing clear assessment of the rate of deterioration. Similarly, the surgical morbidity rate largely varies according to the type of the lipoma. It is widely accepted that lipomas with dural defects are burdened by a higher incidence of wound complications (10–20%), but these data are difficult to extrapolate from mixed series. On the other hand, the present experience reinforces the indication for surgery in asymptomatic children with noncomplex LLs and a tethered cord. An argument in favour of conservative management can be made based on the absence of symptoms in these instances. However, in our opinion, the low number surgical risks and the potentially severe complications in cases of sudden deterioration justify a more aggressive approach. Such an approach seems to be particularly appropriate when a progressive syringomyelia is evident on an MRI scan. Finally, intradural LLs with or without dermal sinus tracts and a normally located conus remain a matter of discussion. Because of the risk of deterioration even in cases of a simple lipoma of the filum (although rare) [20, 21] and the potentially dangerous infection-related complications of intradural dermal sinus tracts, these lesions should also be regarded as possible indications for surgery. Based on the present experience and the review of the literature, we suggest surgery for individuals with dermal sinus tracts (risk of infections) and a clinical and instrumental long-term follow-up for those with a lipoma of the filum. For the latter, however, it is mandatory to exclude the presence of premonitory symptoms/signs through an accurate work-up including physical, neurological, orthopaedic, urological and radiological work-up.

Sudden neurological deterioration is a rare but severe, adverse event that should be taken into consideration with LLs. The acute deterioration often results from infection-related complications of dermal sinus tracts, where the systemic component is predominant, and the neurological deficits can be absent. “True” sudden neurological deterioration remains very rare but raises the problem of prophylactic surgery in asymptomatic patients with radiological implications (a tethered cord and syringomyelia).

## Data Availability

All data generated or analysed during this study are included in this published article.

## Abbreviations

LL: Lumbosacral lipomas
MRI: Magnetic resonance imaging
